# Genome-Wide SNP and STR Discovery in the Japanese Crested Ibis and Genetic Diversity among Founders of the Japanese Population

**DOI:** 10.1371/journal.pone.0072781

**Published:** 2013-08-21

**Authors:** Yukio Taniguchi, Hirokazu Matsuda, Takahisa Yamada, Toshie Sugiyama, Kosuke Homma, Yoshinori Kaneko, Satoshi Yamagishi, Hiroaki Iwaisaki

**Affiliations:** 1 Laboratory of Animal Breeding and Genetics, Graduate School of Agriculture, Kyoto University, Kyoto, Japan; 2 Department of Agrobiology, Faculty of Agriculture, Niigata University, Niigata, Japan; 3 Field Center for Sustainable Agriculture and Forestry, Niigata University, Niigata, Japan; 4 Sado Japanese Crested Ibis Conservation Center, Sado, Japan; 5 Yamashina Institute for Ornithology, Abiko, Japan; Auburn University, United States of America

## Abstract

The Japanese crested ibis is an internationally conserved, critically threatened bird. Captive-breeding programs have been established to conserve this species in Japan. Since the current Japanese population of crested ibis originates only from 5 founders donated by the Chinese government, understanding the genetic diversity between them is critical for an effective population management. To discover genome-wide single nucleotide polymorphisms (SNPs) and short tandem repeats (STRs) while obtaining genotype data of these polymorphic markers in each founder, reduced representation libraries were independently prepared from each of the founder genomes and sequenced on an Illumina HiSeq2000. This yielded 316 million 101-bp reads. Consensus sequences were created by clustering sequence reads, and then sequence reads from each founder were mapped to the consensus sequences, resulting in the detection of 52,512 putative SNPs and 162 putative STRs. The numbers of haplotypes and STR alleles and the investigation of genetic similarities suggested that the total genetic diversity between the founders was lower, although we could not identify a pair with closely related genome sequences. This study provided important insight into protocols for genetic management of the captive breeding population of Japanese crested ibis in Japan and towards the national project for reintroduction of captive-bred individuals into the wild. We proposed a simple, efficient, and cost-effective approach for simultaneous detection of genome-wide polymorphic markers and their genotypes for species currently lacking a reference genome sequence.

## Introduction

The Japanese crested ibis *Nipponia* *nippon* is an internationally conserved bird, listed as “Endangered” in the 2012 International Union for Conservation of Nature Red List of Threatened Species (http://www.iucnredlist.org).

The Japanese crested ibis once flew over much of Japan and northeastern Asia, but overhunting for the feathers and habitat loss devastated their numbers. After the Japanese crested ibis was extinct in Japan, captive-breeding programs have been continued with 5 birds (2 individuals introduced in 1999, 1 individual in 2000, and 2 individuals in 2007) donated by the Government of China, where a very small wild population survived [1]. The current size of the captive-breeding population in Japan is approximately 180, mainly in the Sado Island. The idea of maintaining captive-bred animals for eventual release into the wild is a major aim of modern zoological collections [2], and the Ministry of the Environment of Japan launched a project for tentative release of the Japanese crested ibis on Sado Island in 2008. In April 2012, 3 Japanese crested ibis chicks hatched on Sado Island and became the first of their species borne in the wild in 36 years [3].

Conservation of small or captive populations requires particular concern for the loss of genetic diversity through genetic drift and inbreeding [4]. Knowledge of genetic diversity and structure can be vital to the genetic management of captive populations and reintroduction of captive-bred individuals into the wild. However, it is difficult to obtain precise knowledge of the genetic diversity and structure of the Sado captive population, because there is no pedigree information regarding kinship among the founders. Therefore, to improve management of the Japanese crested ibis toward national project goals, it is important to evaluate the genetic relatedness between the founders by using molecular tools such as single nucleotide polymorphism (SNP) and short tandem repeat (STR) markers. However, information on the genome sequence or polymorphic markers in the Japanese crested ibis remains sparse. Currently available genetic markers include only 26 microsatellites [5–7].

A general limitation of genome-wide polymorphic markers in non-model organisms has been a lack of extensive genomic sequence information from multiple individuals that represent a sufficient portion of the genetic variability of a given population or species. However, next-generation sequencing coupled with restriction enzyme digestion of target genomes to reduce target complexity, such as reduced representation libraries (RRLs) [8,9], restriction-site-associated DNA sequencing (RAD-seq) [10], and complexity reduction of polymorphic sequences (CRoPS) [11], has provided an efficient approach to solving this problem [12]. The Illumina HiSeq2000 sequencing system, released in 2010 provides the highest throughput available, and might enable us to design an efficient, cost-effective approach for the discovery of genome-wide SNP and STR markers.

The aim of this study was to develop a large number of polymorphic markers in the Japanese crested ibis genome by using a combination of RRLs prepared from 5 founder genomes and next-generation sequencing. We also investigated relative genetic similarities between the founders based on their genotype at each marker.

## Materials and Methods

### Reduced representation library construction

Blood samples from the Japanese crested ibis were provided by the Sado Japanese Crested Ibis Conservation Center (Niigata, Japan). Protocols of sample collection were approved by the Animal Research Committee of Niigata University based on conservation project of the Ministry of the Environment of Japan. Genomic DNA samples were prepared from whole blood using the Wizard Genomic DNA Purification kit (Promega) according to the manufacturer’s instructions with slight modification. Blood (60 µl) was washed with 3 mL PBS, and red and white blood cells were lysed with 6 mL Nuclei Lysis Solution (Promega). Aliquots of 80 µg of genomic DNA from each individual were digested with 800 units of *Hae*III or *Mbo*I (TAKARA) overnight at 37°C. Digested DNA was separated by 1.5% agarose gel, and digestion products of 250–350 bp were gel purified using the Wizard SV Gel and PCR Clean-Up system (Promega) according to the manufacturer’s instructions. The *Hae*III- and *Mbo*I-digested fragments were combined and processed as 1 RRL for sequencing on the HiSeq2000 (Illumina). The RRLs were independently prepared from each of 5 founders.

### Sequencing and data analysis

Sequencing was performed at the Hokkaido System Science Co. Ltd. (Sapporo, Japan). Briefly, the combined DNA fragments were end repaired and ligated with the sequence adaptor using the TruSeq DNA Sample Prep Kit (Illumina). The RRLs were distinguished by adding sequence adaptors with different index sequences. The RRLs were pooled and sequenced in a single sequencing lane on the HiSeq2000 for 101 cycles in pair-end mode. Raw data files from the sequencing instrument were deposited in the DDBJ sequence read archive under accession number DRA000585.

Primary data analysis was also performed at Hokkaido System Science. After adaptor trimming with the cutadapt program (http://code.google.com/p/cutadapt/) and discarding reads containing N bases, filter-passed sequence reads from the founder RRLs were divided into 3 groups by their 5′-terminal sequences (both-end *Hae*III, both-end *Mbo*I, and others). Sequence reads within a group were clustered by the clustering program “SEED” (http://manuals.bioinformatics.ucr.edu/home/seed), and consensus sequences of 300 bp (read-pair) were generated. These consisted of forward and reverse 101-bp reads with internal 98 bases of N. Parameters in the program “SEED” were as follows: --shift was 0 and other parameters were defaults. Consensus sequences with depth ≥10 were used as reference sequences for mapping of read pairs from each founder. Consensus sequences used for mapping were deposited in the DDBJ sequence read archive under accession number DRZ002863. Mapping was performed by the short read aligner program “bowtie” (http://bowtie-bio.sourceforge.net/index.shtml). Parameters in the program “bowtie” were as follows: -I was 300, -X was 300, -v was 3, and -- best option was specified.

### SNP discovery, genotyping, and haplotyping

The 123,506 predicted SNPs whose depth was ≥100 were extracted from the mapping results. Putative SNPs were selected by the following filtering processes: (1) Alleles with a depth of 1 in a founder sample at predictive SNP positions were ignored. (2) SNPs with a depth of more than 300 reads in any individual were filtered out(3). If the read pairs for an allele were more than 5% of the total depth from a founder, the corresponding alleles were considered present. Then, SNPs with 3 or more alleles in any individual were discarded(4). After the predictive SNPs with 2 alleles were identified, predictive SNPs for which the depth ratio between the 2 alleles in any individual was more than 3 were also removed. We used the putative SNPs with depth ≥20 in each founder for the following genetic analysis.

For haplotyping, all consensus sequences with polymorphisms at more than 1 position were extracted. Then, a set of SNPs with the same depth within 202-bp consensus sequences was treated as a haplotype (if the difference in depth between SNPs was <4 due to sequence error, they were assumed to have the same depth).

### Analysis for genetic similarities between founders

The numbers of single founder-specific alleles and the heterozygous and homozygous loci were counted in each founder across the putative SNPs with depths ≥20 in each of the founders. Then, the number of loci with the same genotype was also computed. Principal component analysis (PCA) and multidimensional scaling (MDS) were performed using the princomp function with cor=T option and the cmdscale function with default option by setting 1 correlation as the distance measure in R (http://www.R-project.org), respectively, where counts of the major allele for each locus were used to calculate correlation matrix between founders. Hierarchical clustering was also carried out using the R package pvclust (http://www.is.titech.ac.jp/~shimo/prog/pvclust/) to evaluate stability in the clustering results through multiscale bootstrap resampling. We applied “average” and “correlation” options for the method of agglomerative clustering and the distance measure, respectively, and computed approximately unbiased (AU) p-value and bootstrap probability (BP) value based on 10,000 bootstrap replications.

### STR discovery

All consensus sequences containing 8 or more di-nucleotide tandem repeats, 5 or more tri-nucleotide tandem repeats, or 4 or more tetra-nucleotide tandem repeats were extracted. Consensus sequences that were identical other than those in the repeat-sequence region were grouped by self-mapping with the short read aligner program “bowtie”. Using the mapping results described above, we counted the number of read pairs corresponding to each STR allele.

## Results

### Sequencing strategy

To discover genome-wide SNP and STR markers in the Japanese crested ibis, we generated sequences from RRLs using the next-generation HiSeq2000 sequencer (Illumina). We simultaneously obtained genotype data on each of the markers by sequencing the RRLs prepared independently from the founder genomes.

At the time of this study, the HiSeq2000 produced sequences of 150–200 million DNA fragments with 100-bp read length in 1 sequencing lane. This allowed us to analyze DNA fragments from 0.5 to 1 million loci per genome, with 5 DNA samples and a sequencing depth of approximately 30 (5 sample × 1 million loci × 30 depth = 150 million). Pair-end sequencing with 100-bp read length of 0.5–1 million fragments resulted in 100–200 Mb, which was estimated to represent 8–13% of the Japanese crested ibis genome, supposing the genome size is 1.5 Gb based on several entries in the eukaryotic genome size databases [13].

To prepare RRL with the desired number of fragments, we digested genomic DNA with *Hae*III or *Mbo*I and extracted fragments in the 250–350 bp size range from agarose gels. In a preliminary experiment, the number of size-selected DNA fragments by digestion with *Hae*III and *Mbo*I were estimated to be 0.34 and 0.44 million, respectively, from the yield of isolated DNA fragments. We also chose DNA fragments in the 250–350 bp size range to prevent decreasing sequence data by overlapping forward and reverse 100-bp sequence reads from a restriction fragment. Restriction fragments generated by digestion with *Hae*III and *Mbo*I were combined and processed as a single RRL for sequencing.

The RRLs were independently prepared from each of the 5 founder genomes. Each RRL was distinguished by adding sequence adaptors with different index sequences. The RRLs were pooled and sequenced on a single sequencing lane on a HiSeq2000 instrument for 101 cycles in pair-end mode.

### Illumina sequencing results

We sequenced the 5 RRLs in pair-end mode, generating 339 million 101-bp reads (Table 1). The proportion of high-quality bases (≥Q30) over all sequence reads was >92% in every sample. After adaptor trimming and discarding reads containing N bases, the remaining 316 million reads were used for analysis. The number of reads in each founder was 48–72 million (Table 1).

**Table 1:** Amount and quality of sequenced DNA reads.

	Total	Founder				
		A	B	C	D	E
No. of 101-bp reads	339,597,768	51,920,408	63,860,862	78,009,738	73,383,930	72,422,830
No. of bases (Mb)	34,300	5,244	6,450	7,879	7,412	7,315
% of ≥Q30 Bases		92.3	92.3	92.2	92.2	92.2
No. of trimmed reads	316,436,996	48,357,306	59,497,380	72,706,332	68,382,502	67,493,476

% of ≥Q30 Bases: proportion of high-quality bases (≥Q30) in filter-passed bases

Read pairs combined with paired forward and reverse 101-bp reads were divided into 3 groups by their 5′-terminal sequence (both-end *Hae*III, both-end *Mbo*I, and others) (Table 2). The read-pairs within a group were clustered and consensus sequences were created (Table 2). In total, 31,418,852 consensus sequences were created. The number of consensus sequences with depths (counts of read pairs clustered to identical sequence) ≥10 was 465,471, 249,515, and 1,039,807 in both-end *Hae*III, both-end *Mbo*I, and others, respectively. Though different groups could contain a set of overlapping sequences, estimation from the number of consensus sequences with ≥10 depth would mean that the sequence information generated here represented at least 6–10% of the Japanese crested ibis genome (0.46 million for *Hae*III to 0.71 million for *Hae*III+*Mbo*I, multiplied by 202 base yielded 0.09–0.14 Gb).

**Table 2:** The numbers of created consensus sequences and putative SNPs.

	Group			
	Total	Both-end *Hae*III	Both-end *Mbo*I	Others
No. of read pairs	158,218,498	85,131,252	9,024,997	64,062,249
No. of consensus sequence	31,418,852	4,175,097	952,879	26,290,876
No. of consensus sequence (depth, ≥10)	1,754,793	465,471	249,515	1,039,807
No. of consensus sequence (mapping depth, ≥100 in a total of 5 birds)	532,712	294,989	13,353	224,370
No. of putative SNP	52,512	28,764	321	23,427
No. of putative SNP (mapping depth, ≥20 in each of the 5 birds)	32,157	16,334	224	15,599

### SNP prediction

Because no reference genome sequence is available for the Japanese crested ibis, we searched putative SNPs by mapping read pairs from each founder to consensus sequences (depth ≥10) and filtering (see Materials and Methods for criteria). Approximately 70% of read pairs from each founder were mapped (Table 3), resulting in 532,712 consensus sequences with depth ≥100 in all 5 founders (Table 2). Out of the 123,506 predictive SNPs in these consensus sequences, 52,512 (42.5%) putative SNP markers were detected, fulfilling the criteria (Table 2, the list of all putative SNP sites is provided in Table S1). Further, the number of the putative SNPs with depth ≥20 in each founder was 32,157 (Table 2), and these putative SNPs were used for the collection of genotype data. The list of all genotype data is provided in Table S2.

**Table 3:** Mapping results.

	Founder				
	A	B	C	D	E
No. of read pairs	24,178,653	29,748,690	36,353,166	34,191,251	33,746,738
No. of mapped read pairs	16,154,580	20,825,091	26,120,640	23,684,263	22,881,668
% of mapped read pairs	66.8	70.0	71.9	69.3	67.8

As the 4,842 consensus sequences contained multiple putative SNP sites within a 202-bp sequence; their haplotypes were deduced from mapping data (the list of all consensus sequences containing multiple SNP sites is provided in Table S3). Of these, haplotypes could be determined in 4,080 (84.3%) consensus sequences, but not in 762 (15.7%) consensus sequences. The deduced haplotype numbers were 2–4 in most loci (Table 4).

**Table 4:** The number of haplotypes on consensus sequence containing multiple SNP sites.

No. of haplotype per locus	2	3	4	5	6
No. of locus	2,750	1,054	258	13	5

### Genetic similarities between founders

**Figure 1:**
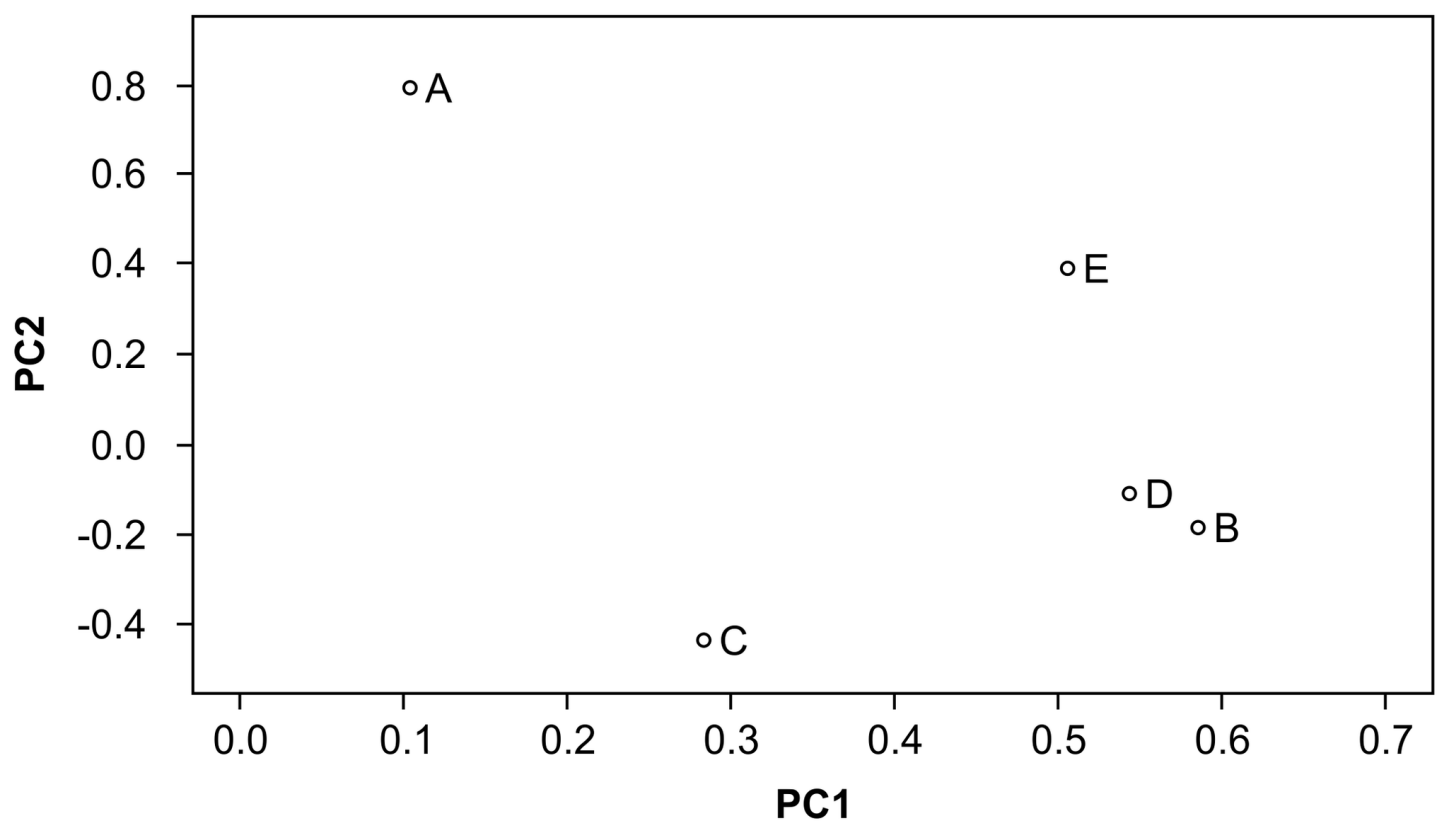
Principal component analysis for the 5 founders by using genotyping data of 32,157 putative SNPs.

**Figure 2:**
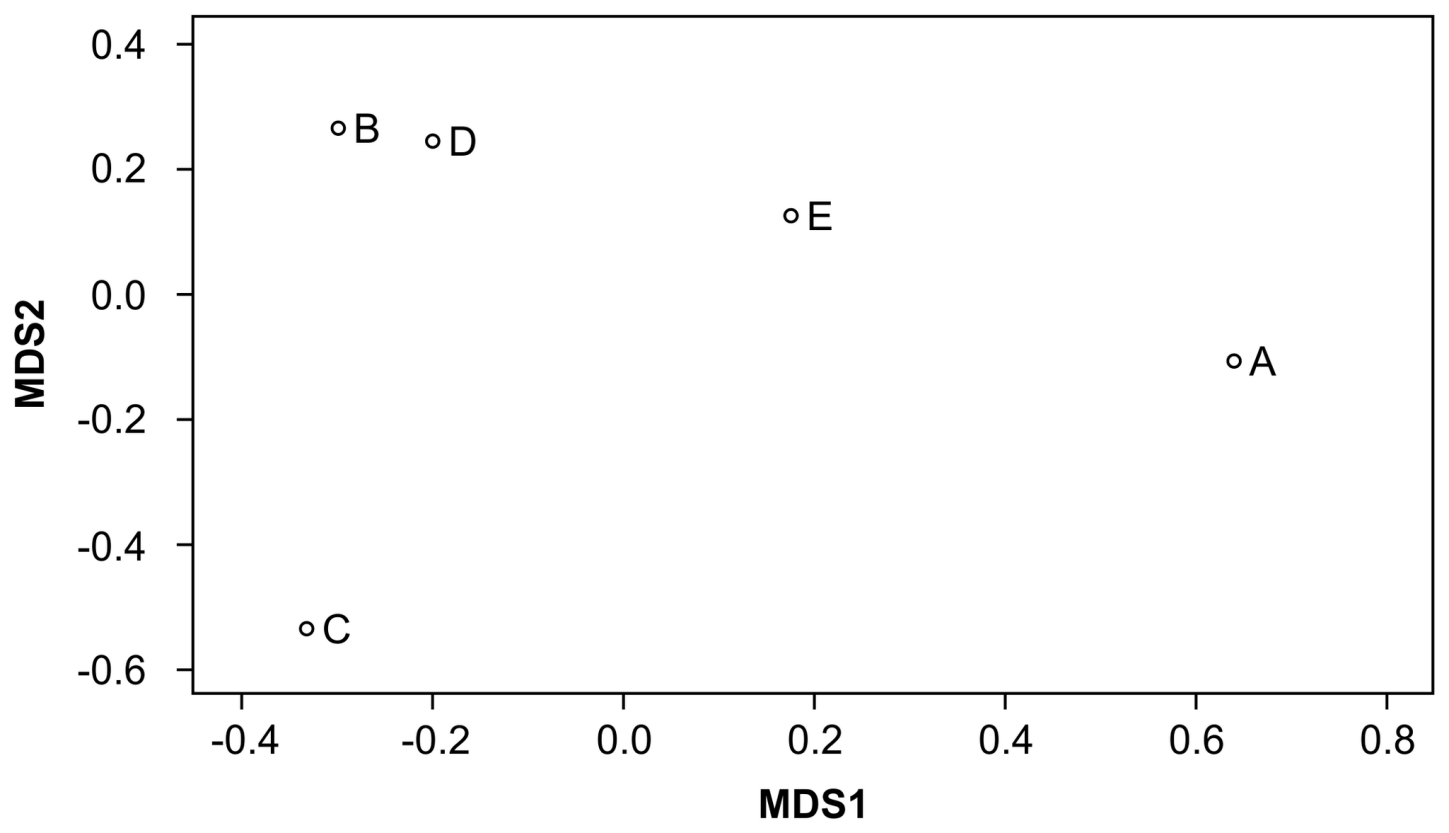
Multidimensional scaling analysis for the 5 founders by using genotyping data of 32,157 putative SNPs.

**Figure 3:**
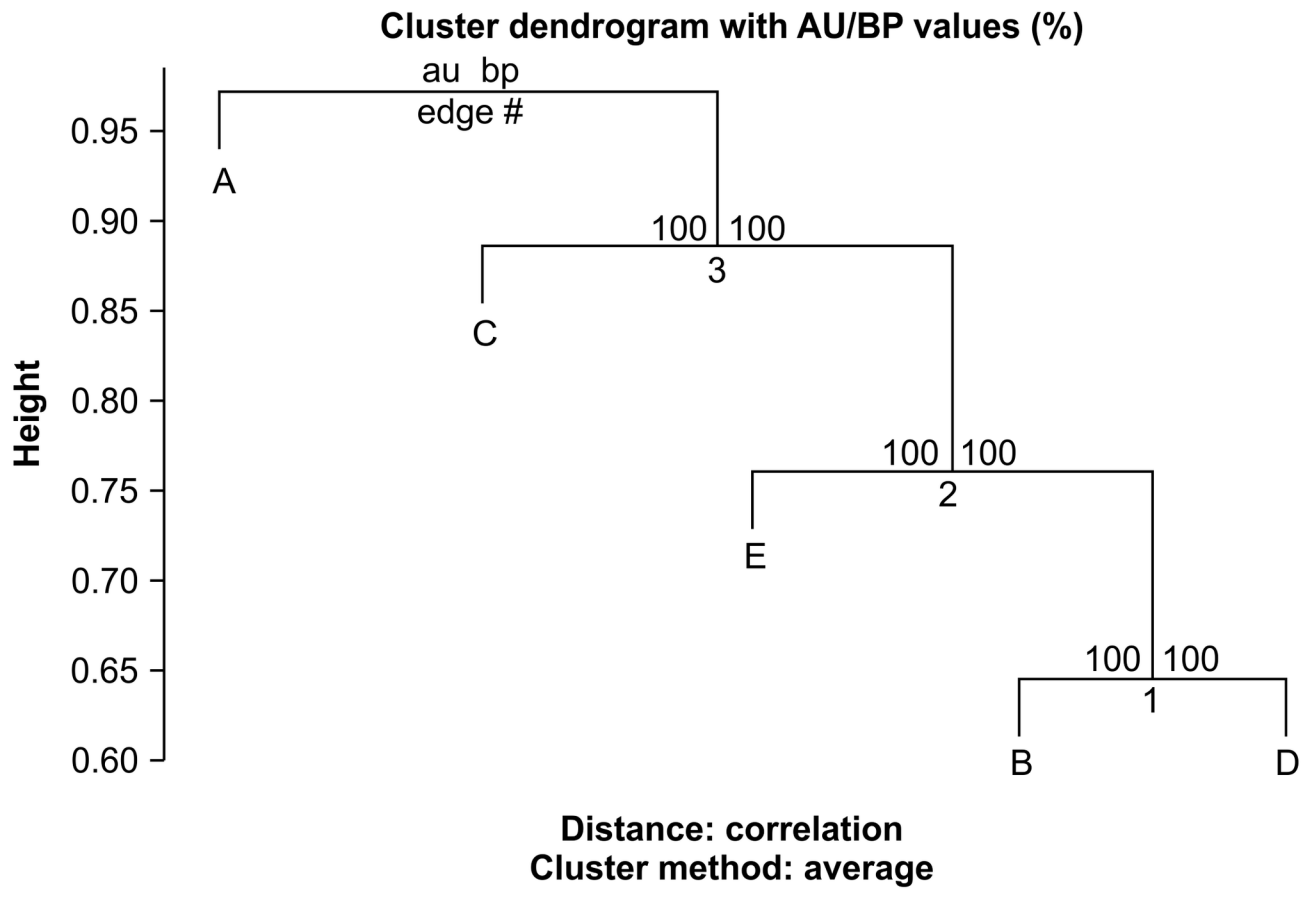
Hierarchical clustering of the 5 founders based on the similarities of their genotype patterns at 32,157 putative SNPs. Values at branch nodes represent AU values (left), BP values (right), and cluster labels (bottom).

The genotype data on 32,157 putative SNPs in each of the founders were used to analyze genetic similarities between them. Single founder-specific allele numbers were 2,087, 1,367, 2,305, 1,676, and 1,003 in founders A, B, C, D, and E, respectively (Table 5). Proportions of heterozygous genotypes and of SNPs whose genotypes were common in pair-wise combination were calculated. The proportion of heterozygous genotypes in each founder was 0.49–0.56 (Table 5). The proportion of SNPs whose genotypes were common in 2 founders was 48.5–59.4% (Table 6). Founders B and E had the highest proportion of common genotypes. We performed PCA using the 32,157 SNPs and used the first 2 principal components (PCs 1 and 2) to visualize the degree of relative genetic similarities among the 5 founders, where PC1 accounted for 32.7% of the variation, while PC2 accounted for an additional 23.1%. This analysis revealed that each individual was located in a relatively dispersed position, although founders B and D were plotted relatively closer (Figure 1). The results of MDS and hierarchical clustering (Figures 2 and 3) were similar to the result from PCA, and AU and BP values by a bootstrap procedure indicated that the dendrogram topology was stable (Figure 3). The results of common SNP genotype and the multivariate analyses were slightly inconsistent owing to the difference between genotype sharing and allele sharing, but seemed to indicate that the genomes of founders B, D, and E shared significant similarities.

**Table 5:** Single founder-specific allele and SNP genotype.

		Founder				
		A	B	C	D	E
No. of specific allele		2,087	1,367	2,305	1,676	1,003
Homozygous for the major allele		12,871	14,504	13,613	15,684	15,506
Homozygous for the minor allele		1,185	462	1,450	785	610
Heterozygous		18,101	17,191	17,094	15,688	16,041
% of heterozygous genotype		56.3	53.5	53.2	48.8	49.9

**Table 6:** Pairwise comparisons of common SNP genotypes between founders A, B, C, D, and E.

Founder					
Founder	A	B	C	D	E
A	-	16,715	15,611	15,931	19,034
B	52.0	-	16,403	18,682	19,095
C	48.5	51.0	-	16,112	16,410
D	49.5	58.1	50.1	-	17,002
E	59.2	59.4	51.0	52.9	-

The numbers (above the diagonal) and the frequency (below the diagonal) were shown.

### STR prediction

To detect STR markers, we extracted all consensus sequences containing 2-, 3-, or 4-nucleotide tandem repeats; we detected 162 putative STR markers, of which 155 STRs were 2 allelic and only 7 STRs were 3 allelic (all putative and all genotyped STRs are listed in Tables S4 and S5, respectively). The numbers of single founder-specific alleles at the 86 STR markers genotyped in every founder were 8, 4, 8, 2, and 2 in founders A, B, C, D, and E, respectively (Table S5).

## Discussion

The Japanese crested ibis *Nipponia* *nippon* is a critically threatened species and an internationally conserved bird. The Ministry of the Environment of Japan has been engaged in a captive breeding and hopes to release the Japanese crested ibis on Sado Island. Whereas genetic management is critical for these projects, information on the genome sequence or polymorphic markers remains sparse. Currently available genetic markers include only 26 microsatellites [5–7].

Several methods of next-generation sequencing coupled with restriction enzyme digestion to reduce target complexity have been developed for the discovery of genome-wide genetic markers, such as RRL [8,9], RAD-seq [10], and CRoPS [11]. Next-generation sequencing of RRLs has been effective in identification of SNPs in species with reference genome sequences, such as mallard [8], pig [14,15], and cattle [9], and in species without reference genome sequences, such as the turkey [16] and great tit [17]. In many studies, RRLs have been prepared from pools of DNA samples from multiple individuals, thus allowing the detection of polymorphisms within a population but not for each individual.

Because the current Japanese crested ibis population originated from only 5 founder birds, we aimed to detect genome-wide polymorphic markers and their genotype in each founder at the same time. We developed an approach using next-generation sequencing and RRL. Five RRLs were prepared from each of 5 founder genomes and distinguished by ligating a sequence adapter containing a different index sequence. The 5 RRLs were pooled and sequenced on a single sequencing lane on the Illumina HiSeq2000 sequencing instrument.

Sequence information, including 316 million 101-bp reads (more than 31 Gb) from a single sequencing lane on the Illumina HiSeq2000, was sufficient for the discovery of genome-wide genetic markers, providing an extremely cost-effective approach.

In this study, 52,512 putative SNPs were detected by creating consensus sequences by clustering sequence reads, mapping sequence reads from each founder to the consensus sequences, and filtering the predicted SNPs obtained by mapping. Of these, the 32,157 putative SNPs whose depth was ≥20 in each founder were selected to analyze genetic similarities. As the 4,842 consensus sequences contained multiple putative SNP sites within a 202-bp sequence, their haplotypes were deduced from mapping data (Table S3). Haplotypes could be determined in 4,080 (84.3%) consensus sequences but could not be determined in 762 (15.7%) consensus sequences, suggesting that these sequences represented multiple loci or sequence errors. These results suggest that putative SNPs include a considerable number of false SNPs. However, if 30% of putative SNPs were false, the remaining 70% could provide a sufficient number of markers for genetic management of the Japanese crested ibis population.

Approximately 52,000 putative SNPs (28,764 in both-end *Hae*III) were found in 530,000 of 202-bp consensus sequences (294,989 in both-end *Hae*III) (Table 2). A rough estimation based on this frequency suggested that the whole genome of the Japanese crested ibis contained approximately 700,000 SNP sites. Because approximately 50% of SNP sites were homozygous in a single individual (Table 5), the number of heterozygous SNPs in a single individual was found in approximately 350,000 sites (the SNP map might have an average density of one SNP per 2000 bp). This may be an overestimation because the putative SNPs detected here apparently included a considerable number of false SNPs.

In the whole-genome sequencing of a single giant panda individual (an endangered species), 2.7 million heterozygous SNPs were detected (1 SNP per 750 bp) [18]. This is approximately 1.95 times higher than that estimated for humans (1 SNP per 1450 bp) [19]. In thoroughbred horses, which are derived from a few founders, 0.75 million heterozygous SNPs were detected (1 SNP per 3,000 bp) [20]. The number of heterozygous SNPs in a single Japanese crested ibis might be much lower than that in pandas and humans, and comparable to or lower than that in thoroughbred horses.

In contrast to SNP markers, which are usually biallelic, STR markers are expected to be multiallelic (3 or more alleles). We extracted consensus sequences containing short tandem repeats and detected 162 putative STR markers. Of these, 155 STRs were biallelic and only 7 STRs were triallelic. We detected no STRs with 4 or more alleles. Moreover, deduced haplotype numbers on consensus sequences containing multiple putative SNP sites were 2–4 in most cases (Table 4). The allele numbers in several tens of STRs previously developed were 2-5 in Chinese population and 2 or 3 in Japanese population [5–7]. The only 2 haplotypes in mitochondria DNA control region were detected in Chinese wild and captive populations [21]. Our results obtained using a large number of genome-wide markers supported lower genetic diversity in the Japanese crested ibis populations previously estimated from a small number of markers. It was reasonable that the genetic diversity in Japanese population was somewhat lower than that in Chinese population because 5 founders of Japanese population originated from China.

Unfortunately, because of the absence of a reference genome sequence for the Japanese crested ibis, we could not determine whether putative SNPs and STRs represented polymorphisms at a single locus or multiple loci associated with repeated sequences or gene families. In addition, information about the locations of putative SNPs and STRs on chromosomes or linkage between markers remains unknown. To determine whether the putative SNPs or STRs in this study were true heritable genetic markers, further analysis is necessary.

Although validation of SNP and STR markers has not yet been performed, we thought that the genotype data on putative SNPs or STRs in each of the 5 founders could be useful for analyzing the relative genetic similarities between them. The proportion of heterozygous genotypes in each founder was 0.49–0.56 (Table 5). The proportion of SNPs whose genotypes were common in 2 founders was 48.5–59.4% (Table 6). PCA and MDS indicated that each individual was located in a relatively dispersed position, except for founders B and D plotted closely (Figures 1 and 2). These results suggest that genome similarities were not high. Whereas no pair having closely related genome composition was observed, smaller numbers of 202-bp read-pair haplotypes and STR alleles suggested that the genetic diversity of the population in total was much lower than that expected when they were unrelated (i.e., 10 of maximum haplotype or allele number in 5 birds). Lower genetic diversity in a population might be reflected by a smaller number of alleles and haplotypes at any locus and/or longer linkage disequilibrium, rather than by total number of SNPs in whole genome or proportion of heterozygous genotypes.

The comparison of genotypes at each putative SNP revealed that each of the 5 founders had 1200–2000 potential single-founder specific alleles. The loss of these specific alleles will directly reduce genetic diversity in the population. Therefore, it is important that single-founder specific alleles are passed on to some descendants, increasing the allele frequencies in the population.

The availability of a large number of SNPs and STRs predicted here provides sufficient markers to study the Japanese crested ibis population structure and to develop methods for parentage testing, individual identification, and genetic management. Further analysis of a large number of accurately inferred polymorphic markers will also facilitate the construction of linkage maps of the Japanese crested ibis genome.

In conclusion, this study provided important insight into protocols for genetic management of the captive breeding population of Japanese crested ibis in Japan and will help in extending the national project for reintroduction of captive-bred individuals into the wild.

We proposed a simple, efficient, and cost-effective approach for the simultaneous detection of genome-wide polymorphic markers and their genotype data for species lacking a reference genome sequence. Our proposed approach might be useful for an extremely small population such as an endangered species or a population originating from a small number of dominant founders.

## Supporting Information

Table S1 **Mapping results of 52512 predictive SNPs.**
(XLS)

Table S2 **Mapping results and genotypes 32157 putative SNPs.**
(XLS)

Table S3 **Haplotypes in consensus sequences containing multiple SNP sites.**
(XLS)

Table S4 **Mapping results of 162 putative STRs.**
(XLS)

Table S5 **Mapping results and genotypes of 86 putative STRs.**
(XLS)
